# Unlocking Better Asthma Control: A Narrative Review of Adherence to Asthma Therapy and Innovative Monitoring Solutions

**DOI:** 10.3390/jcm13226699

**Published:** 2024-11-07

**Authors:** Emanuel Poplicean, Alexandru Florian Crișan, Emanuela Tudorache, Patricia Hogea, Roxana Mladin, Cristian Oancea

**Affiliations:** 1 Doctoral School, “Victor Babes” University of Medicine and Pharmacy Timisoara, Eftimie Murgu Square 2, 300041 Timisoara, Romania; emanuel.poplicean@umft.ro (E.P.); roxana.mladin@umft.ro (R.M.); 2 Center of Research and Innovation in Personalized Medicine of Respiratory Disease (CRIPMRD), “Victor Babes” University of Medicine and Pharmacy Timisoara, Eftimie Murgu Square 2, 300041 Timisoara, Romania; emanuela.tudorache@umft.ro (E.T.); hogea.patricia@umft.ro (P.H.); oancea@umft.ro (C.O.); 3 Pulmonary Rehabilitation Center, Clinical Hospital of Infectious Diseases and Pulmonology, “Victor Babes”, Gheorghe Adam Street 13, 300310 Timisoara, Romania; 4 Research Center for the Assessment of Human Motion, Functionality and Disability (CEMFD), “Victor Babes” University of Medicine and Pharmacy Timisoara, Eftimie Murgu Square 2, 300041 Timisoara, Romania; 5 Pulmonology University Clinic, “Victor Babes” University of Medicine and Pharmacy Timisoara, Eftimie Murgu Square 2, 300041 Timisoara, Romania

**Keywords:** asthma, treatment, adherence, inhalers, asthma control

## Abstract

This review addresses the ongoing challenges in asthma management, particularly focusing on patient adherence to inhaler therapy. Asthma, a chronic condition characterized by variable respiratory symptoms and airflow obstruction, can lead to significant morbidity and mortality if not properly managed. Despite advances in inhaler technology and therapeutic options, non-adherence remains a significant barrier to optimal asthma control. This review explores both intentional and unintentional non-adherence, influenced by factors such as age, socioeconomic status, and the complexity of inhaler devices. The Global Initiative for Asthma (GINA) provides guidelines aimed at improving adherence through targeted interventions, and this review examines their application. Common inhaler technique errors, including incorrect inhalation speed, not exhaling before inhaling, and failure to hold breath post-inhalation, are identified as major contributors to inadequate asthma control. Furthermore, the review explores the emerging role of electronic monitoring devices (EMDs), such as CapMedic and DigiHaler, which offer real-time feedback to enhance inhaler technique and adherence. The role of biomarkers in assessing adherence and the potential of personalized treatment strategies, including biologic therapies, are also discussed. Overall, addressing adherence requires a comprehensive approach that integrates patient education, tailored interventions, and technological innovations to achieve better clinical outcomes in asthma management.

## 1. Introduction

Asthma is a chronic, heterogeneous condition characterized by airway inflammation, with symptoms that vary in time and intensity, such as dyspnea, chest tightness, cough, and wheezing [1,2]. The World Health Organization estimated that more than 262 million people were diagnosed with asthma in 2019 [3], and this condition was responsible for 455,000 deaths worldwide; most (67%) could have been prevented [2,4,5]. Higher rates of morbidity, mortality, and asthmatic exacerbations have been observed in non-adherent patients [5,6,7,8,9]. According to the National Review of Asthma Deaths (NRAD), non-adherence to treatment was the most important modifiable factor in preventing deaths [5,10].

Asthma is most diagnosed in childhood, although it can become clinically present at any age [2,11]. According to studies of the specialized literature, asthma is more prevalent in women (10.4%) than men (6.2%), and people with a poor financial situation (11.8%), black breed (10.2%), and Hispanics (14.9%) are more likely to develop asthma [12].

In managing asthma, the correct use of inhaled therapy is a fundamental component that is essential for achieving therapeutic effects and adherence [13]. Although they cannot be prevented entirely, asthmatic exacerbations, regardless of the triggering factor (aeroallergens, respiratory pathogens, and environmental pollutants) [14], can be reduced with appropriate treatment with inhaled corticosteroids (ICS) or ICS/long-acting β-agonists (LABA) [15,16,17,18]. In addition, it has been found that after an asthmatic exacerbation, patients become 20% more adherent to treatment (*p* < 0.001) [19]. The rate of hospitalizations due to asthma is considered to double for every 25% increase in the proportion of time without the use of ICS (relative rate 2.01; 95% CI 1.06–3.79) [9]. According to GINA, up to 70–80% of asthma patients do not use their inhalers correctly, and half of both adult and pediatric asthma patients do not take their long-term medication at least part of the time, leading to uncontrolled asthma [1]. Overall optimum adherence rates remain relatively low, between 22% and 63% [20].

Statistics show that treatment is not followed in chronic diseases as recommended in about 50% of cases [21,22,23,24]. For chronic lung diseases, including asthma, estimates indicate an even lower adherence, ranging from 22% to 78%, probably due to the extra effort required to use inhaled therapies [21]. Other studies estimate that asthma non-adherence in adults and children varies between 35% and 75%, depending on the measurement methods [25]. In addition, daily control inhaler use among asthma patients enrolled in a study conducted in several Asian countries was only 14% [16]. Objective measurements showed that self-assessment overestimated adherence in asthma by more than 50% [26,27]. Still, sometimes they have doubtful acceptability to patients, have a cost disadvantage, and sometimes they can associate technical problems and prove challenging to use [28].

Adherence should be regularly assessed in all patients with asthma. When patients recognize that they are not adherent, they should be supported and not blamed, as it is proven that friendly and empathetic communication will increase patient adherence to treatment. In addition, self-reported non-adherence is an essential qualitative indicator of poor adherence [29].

When assessing the adherence of any pathology, it should be kept in mind that it may temporarily improve if the patient is aware that they are being monitored (“Hawthorne effect”) [30].

The effectiveness of inhalation therapy may be overshadowed by critical handling and inhalation errors that significantly reduce the amount of drug administered, leading to poor control. The increased frequency of these errors has been associated with age, previous training, comorbidities, educational and socioeconomic status, factors strongly correlated with increased exacerbations and care costs in this condition [31,32,33].

Some overview data on adherence in asthma discussed in this review are represented in Table 1.

This review aims to highlight the types of non-adherence and factors leading to non-adherence in asthma and identify the advantages and disadvantages of various adherence monitoring methods and different types of inhalation devices used in asthma in order to improve adherence and combat inhalation technique errors. Literature searches were performed on PubMed, Web of Science, Scopus, and Google Scholar. We used the following MeSH terms for the PubMed search: “asthma”, “asthma treatment”, “asthma adherence”, “inhalers, and “asthma control”. The search was limited to journal articles written in English. Thus, we identified several scientific articles that investigated treatment adherence in asthma patients. In composing this review, we have used both topical studies and other asthma adherence reviews from the last 10 years in the hope that we can contribute to improving clinical outcomes among these patients.

## 2. Low Adherence Factors in Asthma

Among the main reasons for non-adherence are age, adverse effects, cost, patient education, comfort, and satisfaction with using the inhaler device, which is strongly associated with increased adherence and improved clinical picture. Given that the prevalence of asthma is growing among young people, age is a crucial element, as it has been observed that teenagers have a lower adherence rate compared to other age groups [34]. Increasing age was associated with better adherence to asthma therapy. However, difficulties in using inhalers, especially in terms of inhalation techniques, may occur in younger children with asthma [21].

Since 2003, the World Health Organization has defined three types of non-adherence (intelligent non-adherence, erratic non-adherence, and unwitting non-adherence), which should be viewed from different perspectives. The patient characterized by intelligent non-adherence is the patient who deliberately discontinues treatment as a rational decision based on mild symptomatology, cost of treatment, or fear of side effects. Erratic non-adherence is an unintentional form of non-adherence and is often associated with forgetfulness. Unwitting non-adherence is encountered when patients forget or do not understand medical instructions regarding the use of medication, for example, improper inhalation technique [25].

Nowadays, GINA divides the components contributing to suboptimal adherence into several categories: medication-related, intentional, and unintentional [1,21].

Medication-related factors include difficulties with inhalation technique. This is a critical element in the correct administration of asthma treatment [35] and is a vital component of adherence [36]. In addition, cost or fear of adverse effects of the drug may influence adherence, and the use of more inhalers than one or increased frequency of dosing may contribute to suboptimal adherence.

Intentional non-adherence factors are based on individual patient choices, such as the perception that treatment is unnecessary, failure to achieve desired expectations, neglect, or stigmatization. In contrast, unintentional factors are unconscious actions, such as misunderstanding instructions, forgetfulness, or associated comorbidities [19,21,37,38]. Unintentional adherence includes incorrect inhalation technique, which may also be influenced by certain comorbidities such as arthritis, depression, or cognitive impairment [21]. Another study conducted in Saudi Arabia shows a negative correlation between adherence to ICS, anxiety, and depression (*p* < 0.001), emphasizing the importance of early detection of psychological symptoms in these patients [39].

Compared to patients with COPD, where non-adherence is predominantly unwitting, in asthmatic patients, non-adherence was more frequently deliberate and erratic [23,40]. This is often because asthma patients adhere to treatment during symptomatic episodes and less when they are symptom-free. In addition, effective communication between the healthcare professionals and the patient increases engagement and motivates the patient to adhere to treatment. Furthermore, in some situations, friends/family’s opinions, culture, or underlying beliefs may influence adherence to treatment for asthma [41].

A study that quantified the adverse effects of asthma medication by questionnaires shows that 64% of patients treated with ICS and 88% of those treated with oral corticosteroids (OCS) reported at least one adverse event, and these patients had strong treatment-related concerns (*p* < 0.0001), which were associated with poor adherence to treatment. The same study shows that physicians’ estimates of adverse events are up to 5 times lower than those reported by patients [42].

At the same time, the patient’s knowledge, beliefs, perceptions, and expectations are directly related to the patient, and a lack of trust in the doctor will lead to non-adherence. Patients with a lower financial situation may have poor adherence because they will not be able to afford the medication or even have difficulty understanding the instructions for using the inhaler due to stress [34]. In terms of cost, it has been observed that adherence is negatively influenced when the medication offset is lower. For this reason, patients reduce the number of inhaled doses, hoping that the inhaler does not run out very quickly [41]. By reducing co-payments to ICS, it can increase adherence by 5.88% [29]. Also, lifestyle changes and stopping smoking may impact asthma adherence [34].

Proper patient screening and education can prevent critical inhalation technique errors, such as operating devices against the lips, teeth, or tongue. These events lead to increased emergency department attendance, hospitalizations, and overuse of antibiotics and OCS [21]. Lack of inhaler adherence is one of the leading causes of increased asthmatic exacerbations, with both intentional and unintentional non-adherence factors contributing to this situation [43,44].

## 3. Inhalation Devices, Techniques and Challenges

### 3.1. Types of Inhalation Devices and Their Characteristics

Inhaled therapy is the predominant therapy for most cases of asthma. It is used both in the prevention of asthmatic exacerbations and in severe asthma attacks. Therapy is effective with the delivery and action of the medication to the lungs, and systemic side effects can be reduced. Inhaled medications include inhaled corticosteroids, short/long-acting bronchodilators, and anticholinergics [45,46,47,48,49,50,51,52].

In recent years, medical companies have improved the efficiency and performance of inhalers. Non-adherence to medication and incorrect use of inhalers represent significant barriers to optimal disease management of patients with asthma. The importance of the patient’s ability to use the device correctly, and the educational role of the treating physician in this regard, has received more attention [47,50,51,53].

Pressurized metered-dose inhaler (pMDI) devices have been available since the 1950s and come in small, lightweight, pressurized aerosol devices. The device is easy to use but remains one of the most challenging inhalers for patients. The patient has to arm the device, which will release a dose of the medication they will inhale [33,47,49,51,54,55]. The inhalation technique using pMDI devices identified both positive elements, such as increased patient satisfaction, unaided use of the device, improved technique through education, and cost savings by avoiding hospitalization, and negative aspects, such as the need to take a slow and deep breath in for 3–5 s or maintaining apnea for 5–10 s post-inhalation. Actuating two puffs simultaneously is another common error with this device [47,56,57].

Aerosol-type devices (Nebulizers) devices have been available since the late 1970s, and they convert drug solutions into aerosols and deliver them directly to the lungs. They are given regardless of the degree of bronchial obstruction. It is the treatment of choice for respiratory distress. Due to poor solubilization, these drugs can crystallize/precipitate, making nebulization difficult. Unlike MDIs, nebulizers are less effective in delivering precise dose medication. With nebulization, more drug is wasted, the production cost is higher, and the risk of contamination is high. For this reason, compared to nebulizers, MDI therapy provides reliable dosing, delivers accurate and constant doses, and is less costly. Nebulizers should be used in patients who are unable to use other inhalers. Jet, ultrasonic, and mesh nebulizers are currently available categories [47,49,51,58,59,60]. A questionnaire-based study interviewing 99 patients, 103 doctors, and 650 nurses about treatment for acute exacerbations of asthma and COPD in adults shows that 60.6% of patients preferred the nebulizer to the MDI spacer. In addition, 49.5% of physicians and 49.1% of nurses perceived the nebulizer to be more effective, compared to 10.7% and 34.5% for the MDI spacer, respectively [61].

Inhalation chamber (Spacer): This device is commonly used for children or the elderly who cannot synchronize their breathing with the release of asthmatic medication. The mouthpiece of this device allows inhalation of the contents but does not allow exhalation through it. This device can reduce oropharyngeal deposition by 80–90% by slowing the speed of inhaled particles. The accuracy of this technique seems better compared to MDI without spacer. Notably, there is no need for coordination between inspiration and MDI arming, so the user does not need deep inspiration and apnea, and the risk of developing oral thrush is lower. Instead, it was observed that patients may forget to shake the inhaler, some patients may not feel comfortable with the spacer, and the risk of contamination can also be an impediment [33,47,49,58,62,63]. In addition, one study suggests that over 60% of patients continue to misuse the MDI despite using a spacer [33].

Dry powder inhaler (DPI), available since the 1970s, is found in multiple forms, incorporating different drug combinations. An inhalation effort is required for proper administration, which differs in intensity depending on the type of device used. Still, it is not obtained in 26–29% of the cases, being the most frequent technical error in these devices [47,49,51]. It appears that in asthmatic children, DPI devices are more correctly used than pMDI without spacers, according to a systematic review [64].

Turbuhaler device is a cylindrical device, with medication as a very fine, dry powder. It delivered a higher medication dose and showed good pulmonary bioavailability [65,66]. A systematic review of the inhalation technique in asthmatic children, which enrolled 28 studies, claims that the inhalation technique with Turbuhaler and Diskus is better than with pMDI in asthmatic children [64]. However, a study of 96 patients in China showed that 1 in 5 patients (19.8%) of subjects did not use the Turbuhaler device correctly, 61.5% of patients had a good technique with this device, and 18.8% had a satisfactory technique. According to this study, older age, lower level of education, and male gender were associated with the incorrect use of Turbuhaler devices [67]. The emitted dose for both substances in the compound decreases radically when the inhaled air flow rate is less than 60 L/min (*p* < 0.05) [68].

The Diskus is a low-resistance DPI device, and the design of this inhaler has been created to be easy to use [68,69]. Among the main errors in this device, highlighted by a study of asthmatic children, are incorrect inhalation positioning (60%), insufficient deep inhalation (18%), exhalation into the device (15%), and failure to open the inhaler correctly. The same study notes that the quality of Diskus administration after the first instructions decreases rapidly and progressively at home, even though the technique has been overestimated at hospital visits [70].

Nexthaler is an extra-fine DPI with several unique and original features that provide feedback to the patient on the inhalation process. The system delays drug delivery until the patient’s inspiratory flow rate is adequate (>35 L/min). This process reduces the emission of large particles (>5 microns), increases the fraction of fine particles inhaled, and favors drug delivery to the lower respiratory tract. It is easy to use, demonstrates better patient satisfaction than other DPIs, and does not require many trials to achieve error-free use [51,71,72,73,74]

Forspiro is another DPI device. The device is patient-approved, intuitive, and easy to use [69,75]. The ASSURE study was designed to assess the control of bronchial asthma, patients’ quality of life, and their adherence to Forspiro treatment. Two hundred four patients used the Forspiro device twice daily; of these, 152 (74.5%) of the patients were satisfied with Forspiro, and 95 (46.6%) of patients assessed their symptoms as “improved”. In contrast, 76 (37.3%) patients reported no changes in symptoms. Adherence was rated as better with the study device than with previous treatment in 64 (31.4%) patients, the same in 91 (44.6%), and worse in 6 (2.9%). The technical aspects of the device (easy of use, low weight, ergonomic design, and mouthpiece shape) satisfied 65% of the patients. About 140 (68.6%) of the patients reported that Fospiro helped them to adhere to the prescribed treatment, while 16.2% reported that the device aspects did not help to increase adherence [75].

**Figure 1:**
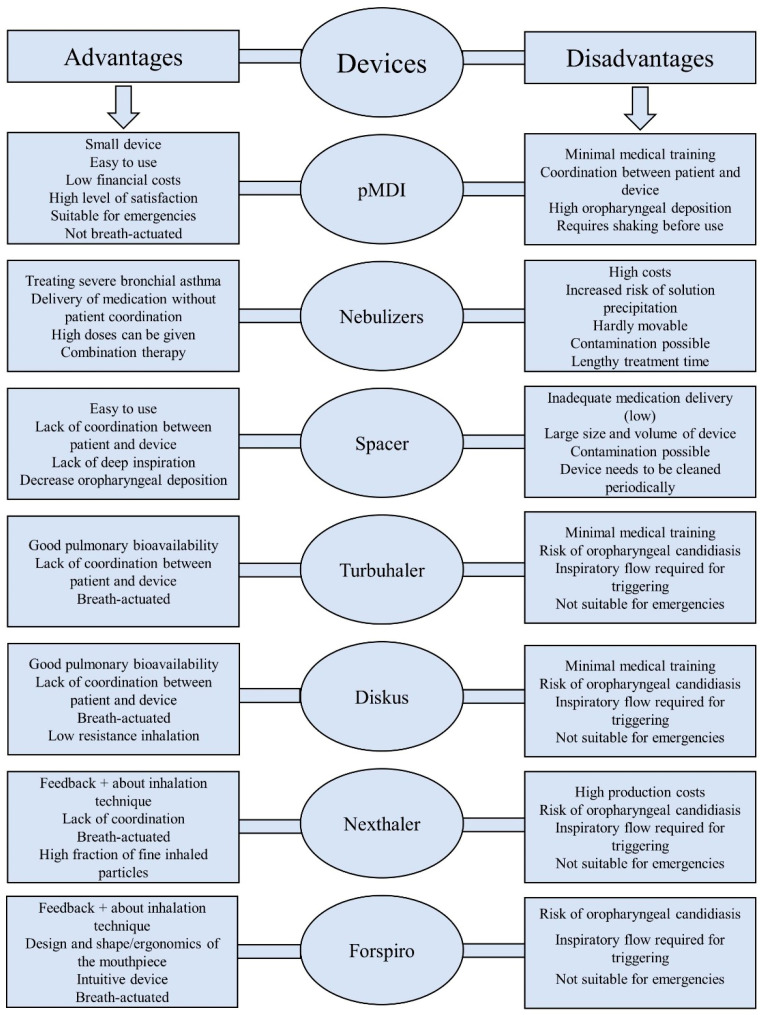
Advantages and disadvantages of current inhalation devices.

The advantages and disadvantages of the most representative inhalation devices discussed in this review are represented in Figure 1.

### 3.2. Difficulties in Using Inhalers

Inhalation therapy remains one of the most complicated self-administered therapies, and this aspect is still underappreciated [47]. The evolution of inhaler devices is fulminant, yet current data suggest that this does not lead to clinical efficacy and demonstrable improvements but rather increases in patient satisfaction [50]. In daily respiratory practice, the most used inhalation devices for aerosolized medication delivery are the pressurized metered dose inhaler (pMDI) and the dry powder inhaler (DPI) [31,32,49,76]. Patient behavior may also be influenced by device design [69].

Multiple studies show that 50% to 94% of patients have poor inhalation techniques [32,77,78,79,80]. Thus, although direct intrapulmonary treatment administration is attractive, the goal is not achieved due to the rich alveolar region that facilitates rapid absorption [81].

A study published in 2018, conducted on 92 asthma patients, Mishanding of pMDI and DPI inhalers in asthma and COPD, describes the most common errors asthma patients experience. For pMDI users, these were holding the breath after inhalation (60% of patients succeeded during the first inhalation, 50% succeeded during the second inhalation), inhaling too quickly and too strongly (52%, 61%), failure to exhale correctly before the next inhalation (48%), and no shaking of the pressurized reservoir before use (48%, 43%). Among the DPI users, these were inhaling too slowly and insufficiently strongly (38%, 36%), prematurely stopping inhalation, or inhalation time was too short (less than 3 s) or absent. These observations demonstrate the importance of the technique of device use to facilitate and control asthma more effectively. Early cessation of inhalation during MDI use (*p* = 0.016), nasal inhalation during MDI actuation (*p* = 0.002), and lack of exhalation before inhalation from the DPI (*p* = 0.027) were the most common errors found between the two groups. In this study, 92% of patients made at least one inhalation error [78].

DPIs require a quick and forceful inhalation for correct administration, compared to MDIs and nebulizers, where this inhalation is unnecessary [47,49,81].

Compared with DPI, in pMDI inhalers, patients may have difficulty coordinating between actuation and inhalation, especially in children and the elderly. In contrast, with DPI, patients may have trouble inhaling strongly enough to activate the device, so the inhalers will not be used correctly [21,31,47,49,58,80,81]. For this reason, DPI is associated with better technique in cognitively impaired patients and greater competency than MDI [33,82].

A meta-analysis shows that between 14% and 92% of patients make at least one critical error and quantifies the percentage of critical errors in inhalation technique according to the device as follows: for MDI 45.6% [95% CI 26.0–66.6] and for DPI the rates were highly variable: Diskus 20.8% [95% CI 13.7–30.2], Turbuhaler 40.1% [95% CI 28.6–52.9], Aerolizer 14.2% [95% CI 11.0–18.1], and Handihaler 42.4% [95% CI 28.8–57.1]. The overall error rate (critical and non-critical) for MDI devices was 86.8% of patients [95% CI 79.4–91.9] with at least one error, and for DPI, the overall error frequency was 60.9% of patients [95% CI 39.4–79.0] [32]. Another study describes comparable results, accumulating errors in inhalation technique to 84.2% for MDI and 51.9% for DPI (*p* = 0.013) [80].

The CRITIKAL study, which included data from 3660 patients, showed that inhalation was insufficiently slow and deep in 47.2% of patients who used an MDI and insufficiently fast and forceful in 32.1% of patients who used a Turbuhaler and 38.4% of patients who used a Diskus. Not exhaling before inhalation was a common error. This was found in 26.2% of cases in the Turbuhaler cohort, 32.4% in the Diskus cohort, and 25.4% in the MDI cohort. Also, 49% of patients using Turbuhaler made “twisting errors”. In this study, the fewest inhalation errors were recorded in the Diskus cohort [83].

For the correct use of an inhaler, the patient’s age and comorbidities must be considered. In a study on 62 patients, the strength of the fingers in handling the inhaler was evaluated, and various difficulties in administering the treatment in patients with rheumatic diseases were observed, which may affect their adherence [79].

A study of 171 pediatric patients showed that 68.1% of patients who used MDI for asthma treatment used the inhaler correctly, while the percentage for patients who used DPI was only 34.6% (*p* < 0.001). Also, patients who had been instructed three times before use operated the inhaler more correctly (*p* < 0.001), and in these patients, asthma was better controlled (*p* < 0.001). In addition, a higher frequency of incorrect inhaler use was observed in patients with mothers with a lower degree of education (*p* = 0.007) [84].

Another prospective observational study, which assessed the need for instruction to minimize inhalation technique errors, concludes that at least three repetitions of instruction are needed to achieve correct inhalation skills (no errors, or less than 10% errors in total), in more than 90% of enrolled patients with asthma or COPD. In this study of pMDI patients, the most common inhalation error was device manipulation (49%) due to poor coordination. In comparison, in DPI, it was inhalation modality (60%), such as no exhalation before inspiration, unforced inspiration, or no breath-hold at the end of inspiration. In the case of the Elipta (a DPI device), all enrolled patients thoroughly learned the maneuver after three training sessions, and this device has the lowest inhalation error rates in this study [52].

A systematic review of the literature evaluated inhalation technique errors for MDI. It showed that 86.7% of patients made at least one error, 76.9% of patients got more than 20% of the inhalation maneuver steps wrong, and the most common errors being the maneuver of exhaling completely before inhalation (65.5%), followed by hold breath for 5–10 s (41.9%) and inhale slowly and deeply (39.4%) [33].

When one inhaler is exchanged for another, or two or more different types of inhalers are used concurrently, asthma control may be decreased due to confusion about inhalation techniques between devices [47,85], and the direct and indirect medical costs are higher [58]. In addition, when referring to the number of substances administered, it has been observed in some studies in the USA and Japan that in asthma patients on triple closed inhaled therapy in a single inhaler, both adherence, through higher mean number of days covered, and persistence were higher compared to triple multiple inhaler therapy (MITT). Also, a high rate of medical resource utilization (HRU) and exacerbation rates associated with MITT were observed in the US study [86].

Adherence also correlates with the number of treatment administrations [50]. For example, a real-world study showed that adherence was significantly higher (61%) among patients using ICS once daily compared to those using ICS ≥ 2 times daily (41%) [87].

### 3.3. Patient Adherence to Inhalation Devices

Treatment with ICS has been the primary treatment for asthma for a long period, maintaining symptom control, reducing exacerbations, and preserving patients’ quality of life [46,48]. No inhaler device is perfect; inhaler selection should be individualized, considering disease severity, age, patient preferences, and patient satisfaction [47]. Approximately 60% of asthma patients preferred an active or collaborative role in therapeutic decisions [25]. However, the preferred inhaler does not guarantee fewer technique errors, according to a cross-sectional study of 301 adult patients diagnosed with asthma and COPD [50]. Also, the excessive use of β2-agonists is inappropriate, and the concomitant use of β-blockers or non-use of ICS as primary therapy in asthma is a significant error that should be categorically avoided [45].

According to GINA, severe asthma accounts for less than 10% of the total asthmatic patient population, but it is the costliest, representing the most refractory population to ICS treatment [88,89]. Secondary adherence rates to ICS in children and adults are around 50% but can change over time. For example, a study of 163 children showed that adherence to inhaled budesonide was 70% after 6 months and decreased to 60% at one year [29].

Compared to oral or injectable asthma treatments, adherence to inhaled therapies is the lowest [34]. Less than half of asthma patients renew their prescription for an inhaled corticosteroid, and it is estimated that only 8% to 13% of asthma patients continue to renew their inhaled corticosteroid prescriptions after one year. In addition, there was an 11% decrease in the risk of asthma exacerbation for every 25% increase in adherence to treatment [26].

MDIs and DPIs are the most used inhalation devices [31,90]. Compared to MDI, DPI does not require coordination of inhaler actuation with inspiration, which is an advantage for certain patients [33,91]. There are over 200 drug–inhaler combinations available globally [58,90,92,93]. For this reason, each device requires a specific inhalation technique to deliver the optimal drug dose. For MDI, the timing of actuation and slow, deep inhalation with breath-hold after inhalation is essential for the inhalation technique. Delayed actuation of the MDI reduces drug delivery to the lung and increases mouth deposition, and early onset before inhalation predisposes to drug loss in the device [33,54]. DPIs are breath-activated, and most devices require a quick, forced inhalation maneuver for drug delivery [49,81].

When used correctly, these devices have no difference in results [90,94]. In daily practice, poor inhalation technique in patients with bronchial asthma (both MDI and DPI) is significantly associated with poor disease control. This again emphasizes the importance of improving inhaled medication management, leading to decreased side effects [52,93,94].

It is estimated that at least 50% of asthmatics fail to take their maintenance therapy as instructed [95,96]. A study published in 2021 compares ICS adherence with MDI and DPI devices with placebo. Average daily adherence rates were higher in the ICS-treated group (80.9%) than in the first month of the placebo (69%). However, the corticosteroid-treated group’s adherence gradually decreased over the next 5 months [95].

Adherence to treatment remains a significant problem in asthma management. A study of 2598 subjects comparing adherence to ICS treatment between a group using a combination of inhaled corticosteroids and Ꞵ2-long-acting agonist (LABA) other than formoterol (F) and a second group treated with ICS and formoterol (F) shows that adherence was higher in the first group (ICS + LABA) 75.1%, compared to the second group (ICS + F) 59.3% (*p* < 0.03). However, asthma control and work productivity were similar between the two groups. However, it was also observed that in the ICS + F group, the use of weekly Ꞵ2-short-acting agonists (SABA) was lower [97].

Another study evaluating persistence, i.e., the time from inhaler initiation to discontinuation of therapy, conducted in Germany on a total of 11,774 patients receiving inhaled salmeterol/fluticasone propionate inhaled therapy with Forspiro (5887 patients) and Diskus (5887 patients) shows a statistically significant (*p* = 00.1) superior persistence in Forspiro as demonstrated by the overall survival experience of the two populations (12-month study period) [69].

In addition, a study conducted in the Netherlands, which compared the use of Diskus and Autohaler (a pMDI device) at home using videos, carried out on 27 asthmatic children, shows that the most common error in the case of Diskus was incorrect positioning of the device (n = 271; 60% of errors with the Diskus) while in the case of Autohaler insufficiently deep inhalation (n = 39; 62% of errors with the Autohaler). The same study showed that the percentage of days of correct administration was 44% for Diskus compared with 96% for Autohaler (*p* < 0.001) [70]. Counseling children on the correct inhalation technique is associated with improved inhaler technique [64].

More than that, another study of 8000 patients published in 2016 shows that less than 50% of patients report that healthcare staff has not assessed their medication administration technique in the past year. This lack of supervision and correction of inhalation techniques is associated with increased errors. These worrying statistics underline the role of the specialist in implementing proper medication inhalation techniques and practicing repetitive training, which is essential to reduce hospitalizations and asthmatic exacerbations. About 80% of patients considered their asthma controlled, but two-thirds did not recognize an asthmatic exacerbation; 55% of those who considered their asthma controlled had symptoms during usual activities; 52.5% reported nocturnal awakenings due to asthma; and 19.5% of those who did not recognize an asthmatic exacerbation reported an emergency department visit [98].

The bronchial asthma specialist’s priorities are to increase patient adherence to treatment by informing and educating the patient periodically and reassessing and adjusting techniques where the patient is experiencing difficulties [52]. The current trend is to replace inhaled therapies with immunotherapies, which are much safer, more effective, and last longer to keep symptoms under control. However, this step should not be taken until we have ensured that the patient’s adherence and inhalation technique are correctly mastered.

## 4. Adherence Monitoring in Asthma

### 4.1. Asthma Adherence Monitoring Questionnaires

Among the most preferred methods of monitoring adherence in asthma is self-assessment through various questionnaires, mainly due to low cost and high clinical applicability. They are subjective measurement methods, prone to recall bias, and may hide the truth due to fears of not living up to the interviewer’s expectations [22]. However, it has been shown that results are often overestimated by this method compared to other objective measurements [26,30,96].

The TAI (Inhaler Adherence Test) questionnaire has been developed to monitor and improve specialist-mandated therapeutic regimens. The questionnaire consists of 12 items divided into two parts: one is dedicated to the patient, and the second is to the health professional. The basic idea behind developing the TAI questionnaire was to identify the non-adherence and, simultaneously, the barriers asthmatics face in inhalation therapy [6,16,23]. A group of 816 patients participated in a study on asthma treatment adherence. About 28.6% of patients were non-adherent according to pharmacy refill rate (PRR) versus 58.1% according to TAI. When both tools were combined, the percentage of non-adherent patients increased to 64.4% (*p* < 0.001) [6].

Although initially designed to measure adherence in hypertension, the Morisky–Green scale or test has been adapted to assess the use of inhalers, with a sensitivity of 67% and a specificity of 76% [23]. A comparison of these two tests shows that TAI has a statistically significantly better correlation with adherence, as demonstrated by evaluations with electronic adherence monitoring devices (Smart-inhalers) [23,99].

The validity of the 8-item Morisky Medication Adherence Scale (MMAS-8) was also quantified in asthma by a cross-sectional study of 208 patients concerning the Asthma Control Score (ACT) and the Saint George’s Respiratory Questionnaire (SGRQ), which assessed quality of life. MMAS-8 was significantly associated with asthma control and quality of life, with 53% of patients having high, 23% medium, and 24% low adherence to inhaled therapy. In addition, the odds of asthma control increased 1.7-fold for each category (OR 1.65, *p* = 0.027). The SGRQ score was 6.1 and 5.3 points lower in patients with high and medium adherence, respectively, compared with patients with low adherence [9].

Another study, conducted in Japan of 85 adult patients with moderate–severe asthma using the Adherence Starts with Knowledge 20 (ASK-20) questionnaire, defined poor adherence as skipping ICS therapy or failing to recognize the need for ICS treatment daily. Although they had ongoing symptoms, 22% of patients had poor adherence, 78% stated that they never or rarely skipped treatment, and only 4% recognized that they did not take their inhalers more than three times a week. This study shows that the younger age of asthma diagnosis was an independent and significant risk factor for poor adherence to inhaler therapy, as they did not recognize the importance of adherence to medication dose since childhood. ASK-20 total score was associated with ICS adherence (*p* = 0.048) [100].

### 4.2. Complementary Adherence Monitoring Methods in Asthma

A systematic review that studied treatment adherence in children with severe asthma suggests that appropriate interventions can increase adherence from 28–67% to 49–81%, depending on the target group, method, and duration of intervention. Intervention methods studied include home interviews and audio-recorded medical visits, educational interventions, the use of electronic monitoring devices, the introduction of self-monitoring calendars, interactive websites, individualized care schedules, or text messages for dose reminders [28].

Among adherence assessment methods, besides self-assessment questionnaires (37.8%) such as the validated Medication Adherence Report Scale for Asthma (MARS-A) or the Medication Intake Survey—Asthma (MIS-A), other methods such as prescription tracking (32.8%) or electronic dose recording (19.3%) are gaining more and more popularity lately [21]. This is underlined by the fact that for more than 20 years, researchers have suggested that quantifying adherence based on self-assessments is inaccurate and can fictitiously increase adherence up to 50% [26,27,96], so researchers are now turning to other methods to quantify adherence.

A prospective 24-week telephone-followed adherence assessment study in China, involving a total of 1582 patients, shows that although initially, adherence was 83.3% at week 4, it dropped to 42% at week 24 when it was observed that the adherence rate of newly diagnosed patients was significantly lower than those with a known history of asthma, 22.9% vs. 63.9% (*p* < 0.001). The same study shows that the 30–39 age group was the most poorly adherent to treatment (27.3%), women were less adherent than men (38.3% vs. 45.6%), and the most common reason for non-adherence cited by patients was “relief of symptoms after short-term use of controller medication” (43.8%) [101].

Another commonly used method is prescription monitoring. However, Jochmann et al. showed that although patients filled their prescriptions 100% of the time, objective methods such as using electronic monitoring devices showed an adherence varying between 27% and 99%, emphasizing the inconsistency between methods [96,102].

Another option for monitoring inhaler adherence is to track the integrated dose counter, which has multiple limitations, such as the inability to demonstrate correct and actual inhaler use. However, the lack of a battery has the advantage of excluding the risk of electronic failure. In addition, some inhalers, such as the Nexthaler, have better monitoring validity by this method, as only by inhalation will the inhaler meter evolve [30].

Electronic reminder systems can also improve patient adherence to asthma medication [44,103,104,105]. A systemic review shows a weak to moderate impact with these systems, with diminishing effects after 4 weeks of use, reflecting habituation to reminders, especially if the reminder is automated [103,104]. A meta-analysis using financial incentives showed positive benefits for patient adherence. Giving short-term financial incentives (3 months) resulted in beneficial behavior maintained after the incentive was withdrawn [103,106,107].

Gamma scintigraphy, magnetic resonance imaging (MRI), computed tomography, and fluorescence imaging are in vivo methods that can produce images that objectively confirm the amount of inhaled particles in humans [47]. However, these methods are costly, may expose the patient to high doses of radiation, and are not easily reproducible, so their role in monitoring inhalation adherence is almost non-existent for now, being used instead in scientific research.

Self-assessment of adherence through self-report questions or diaries is often overestimated by the patient, with studies showing that objective methods such as electronic monitoring devices (EMDs) show that self-reporting is less accurate [29,96].

### 4.3. Electronic Adherence Monitoring Devices for Asthma

Technology has been called upon for the fairness of the inhalation technique and the communication of feedback to the patient or clinician about the administration. To this end, new electronic monitoring devices (EMDs) capable of quantifying adherence and objectively verifying inhalation techniques have been developed [22,44,47,90,108,109]. The most common devices on the market today are the CapMedic, Digihaler, Hailie Senzor, Inhaler Compliance Assessment, Respiro, and Smart AeroChamber [90].

The CapMedic electronic device includes a sensor that attaches to the MDI. The sensor can be connected directly to the MDI or with the spacer. The sensor records the correctness of the technique and the amount of medication delivered by the device and transmits the collected data to the CapMedic application [110]. The device transmits real-time audio–visual feedback to the patient on errors identified during the technique, such as the number of shakes before use, the orientation of the inhaler during use, that is, angle to upright position, coordination between the patient and the inhaler, inhalation duration, and data on end of inhalation apnea [111]. It can also measure lung function by measuring inspiratory flow and FEV1 (forced expiratory volume in the first second). All these data are recorded by the CapMedic app, and patients can keep a symptom diary. The device is rechargeable and has an autonomy of 200 inhalations or about 100 days of use [90]. In a study of 23 patients in Houston, Texas, CapMedic showed that all patients made at least one error using MDI, and 74% made at least three errors [110].

DigiHaler is a multi-dose digital dry powder inhaler with an electronic module that gives patients and pulmonologists feedback on inhalation techniques and treatment adherence [112]. A study that enrolled patients with uncontrolled asthma showed that in patients who used the DigiHaler system, the likelihood of improved asthma control was 88.7% [113]. One hundred fifty participants with an average age of 24.5 years participated in another study in which PIF (peak inspiratory flow rate) and inhaled volume (inhV) were measured by the DigiHaler and by an inhalation profile recorder (IPR) of another device. The PIF for the DigiHaler was 70.62 L/min and 72.55 L/min for the IPR. Mean percentage differences between DigiHaler and IPR measurements ranged from 2.97% in adults with COPD to 0.16% in children with asthma. The results show a strong correlation between the PIF measurements recorded by DigiHaler and those from the IPR (*p* = 0.02) [114].

The Hailie sensor is an electronic device that can assess inhalation techniques by monitoring inspiratory flow, inhaler agitation, and correct inhaler orientation [115]. Data can be uploaded to the patient’s phone using the Hailie app [48]. This device is capable of recording both inhalation and actuation [115].

The Respiro sensor can be attached to the pMDI, Nexthaler, Elipta, or Spiromax. It can provide feedback on inhaler use. Data are uploaded to a phone or laptop. The app can provide feedback on the correctness of the technique and guide patients to improve the technique. Battery life is 16 to 18 months [90].

Guidelines recommend using the MDI with the spacer for better coordination between the patient and the device [33,54,116]. An AeroChamber electronic medical device was developed to evaluate this technique. Information about inhaler use is stored on an SD card that can be visualized and interpreted. Digital AeroChamber is a rechargeable spacer that uses a sensor to measure the patient’s inhalation flow rate. It can detect common errors in inhalation techniques, such as no inhalation, delayed inhalation, inadequate inhalation volume, and multiple device actuation [116,117]. A study of 42 asthma patients conducted in the Netherlands showed that in the group that used this smart spacer, inhalation errors decreased by 26.2%. In contrast, in the group that did not use it, inhalation errors increased by 14.6% [117].

Another electronic monitoring device (EMD) that assesses both patient adherence and inhaler technique (IT) is the Inhaler Compliance Assessment (INCATM) [47,109,118]. A study involving 18 patients, including 12 with COPD and 6 with asthma, found that INCATM significantly improved actual adherence from 30% to 68% (*p* = 0.001) and also showed that the IT error rate was significantly reduced from 51% to 12% (*p* = 0.002). In addition, patients included in the study expressed positive attitudes toward the device, expressing a willingness to reuse such technologies in the future [118].

Electronic monitoring devices (EMDs) can measure factors that influence inhalation techniques. Inhalation technique measurement technology is highly accurate and can provide relevant data for patients who consciously or unconsciously misuse inhaled medication [24]. Patient education on improving inhalation techniques is essential [58], and EMDs can support and tailor this education. Challenges are related to technical device issues, patient acceptance, evidence of clinical effectiveness, sustainability, and cost-effectiveness [24,119].

The sensors used in EMDs have an increased sensitivity. The fundamental difference between the six EMDs outlined above relates to the sensors (whether they are added or incorporated into inhalers). Patients generally want to feel responsible for their treatment. They are enthusiastic about technological intervention in the inhalation technique, but many problems remain unresolved [90]. Patients may be hesitant to share their medical experiences of adhesion and inhalation recording of EMDs with medical staff. They understand the benefits of disclosing this information but feel restricted in sharing it. Sometimes, they want to establish new relationships with the medical team and keep their asthma under control through remote monitoring and discussions. Still, they are concerned about the security and confidentiality of medical data [24,120].

Electronic monitoring devices (EMDs) are increasing due to their ability to provide objective data about patients’ adherence to prescribed medication. The data generated by EMDs can be transmitted to the physician in real time and paint a picture of patient adherence to prescribed treatment [47,90,108,121]. For example, the Propeller sensor can record the date and time of each use, track adherence to therapy in real time, and provide the information remotely via an app, recording up to 3900 events. At the same time, it can provide reminders for missed doses to stimulate adherence [47,121]. About 84% of healthcare providers report benefits from communicating information to the physician in real time. Patient satisfaction with the Propeller sensor is 99%, while 94% of patients get used to the online platform; 92% of patients find the device easy to use [121].

The cost-effectiveness of EMDs in asthma is still poorly evaluated and often impedes their use [115]. This is why EMDs are cost-effective for a subgroup of asthmatic patients, such as those at high risk of poor adherence or those with uncontrolled bronchial asthma [90,96,122]. The monitoring devices, e-health platforms, and related interventions represent the direct costs. Regarding feasibility, some patients, especially the elderly, face many challenges, such as lack of experience with online platforms, fear or dislike of electronic devices, poor ability to learn the device settings, or low motivation [24,47].

These devices can increase treatment adherence and improve patients’ clinical outcomes [24,108]. Another subgroup indicated for EMD is patients in whom inhaled therapy is initiated for the first time. Correct direction from the onset of the disease may prevent faulty techniques and increase treatment adherence from the first application (*p* = 0.03). Statistical results found no significant changes between the two groups followed. An initial period of use of EMDs of about 3 months is enough to provide the necessary information to the physician on whether the patient will have good adherence and adequate asthma control in the long term (*p* = 0.01) [115].

### 4.4. Role of Biomarkers in Asthma Adherence

Disease control must be achieved by measurements of adherence that correlate with specific biomarkers [30,123].

Recent approaches have categorized asthma according to molecular pathways and cellular mechanisms into high T2 (type 2) and high non-T2 endotypes. The discovery of epithelial cytokines (alarmins) in the context of asthma, IL-25, TSLP, and IL-33, has demonstrated that they play a significant role in triggering type 2-high type 2 inflammation in the airways and has the potential to be targeted for the development of next-generation biologic drugs for the treatment of asthma [123,124,125,126]. Biomarkers of T2-high asthma include eosinophils, IgE, periostin, fractional exhaled nitric oxide (FeNO), or urinary leukotrienes [127,128].

Currently, biomarkers are an essential component in the management of asthma; they can be used to assess non-adherence to ICS through tests such as FeNO or OCS, as well as by tests using prednisolone or cortisol. To monitor the dose of inhaled corticosteroids, the number of eosinophils in the sputum can provide additional data [123,127]. In patients with poor adherence, the level of eosinophils is higher in the sputum, the post-bronchodilator FEV1 value is lower, and these patients are more susceptible to being ventilated for asthma. In the future, there is a need to discover new biomarkers through individual measurements of the various cytokines involved, which may provide additional clinical information [127].

However, the first step to demonstrate that improving treatment adherence can influence asthma control is to identify non-adherence, and ideally, compliance should be measured along with these specific biomarkers [30].

The measurement of fractional exhaled nitric oxide has the advantage over other biomarkers that are easy to measure [129]. For example, compliant patients with difficult-to-control asthma can be identified using FeNO, as ICS administration rapidly reduces FeNO in ICS-responsive patients. In contrast, in ICS-resistant patients, the reduction will not be as pronounced, thus differentiating severe from less adherent asthma patients, reflecting that elevated FeNO in severe asthma may indicate suboptimal adherence [30].

### 4.5. The Relationship Between Poor Adherence and Asthmatic Exacerbation

Only one-third of patients perform the inhalation procedure correctly [58,94,130]. Adequate adherence to inhalers is associated with fewer exacerbations, greater symptom control, lower corticosteroid requirements, and lower disease-related mortality [58,96].

It is essential to target and reduce inflammation and bronchoconstriction and prevent airway remodeling [131,132]. Asthma remains uncontrolled in many patients despite advancements in treatment, leading to a higher risk of exacerbations and increased healthcare resource utilization (HCRU). Uncontrolled asthma was reported in 19.8% of patients in GINA treatment step (GS) 2, 44.8% in GS3, 49.3% in GS4, and 58.6% in GS5. HCRU also increased with the rise in GS, highlighting the need for improved patient education and adherence [133].

Numerous studies have shown that when adherence to asthma medication is higher, better symptom control is achieved, and exacerbations are reduced [21,44,96]. It has been observed that when good collaboration with patients and their preferences or beliefs about treatment are considered, asthma adherence can improve, and debilitating exacerbations will be reduced [21,50].

An increase in asthmatic exacerbation was identified in May compared to other months of the year (*p* = 0.004) [134], and not infrequently, seasonality, season, weather, or environmental factors influence adherence to asthma treatment. It is estimated that 44% of affected children have uncontrolled bronchial asthma, and according to the Centers for Disease Control and Prevention (CDC), in 2021, 39.6% of adults and 38.7% of children who are diagnosed with bronchial asthma have reported at least one asthmatic attack in the past year [135]. A study of 249 children shows an even worse situation, reporting that 55.1% of them had uncontrolled asthma, and 40% required hospitalizations or asthma-related medical visits in the past year [136]. These data emphasize that adherence to asthma should be persistent over time, not artificially increased in certain seasons or months, when contact with certain triggers of an exacerbation is anticipated, or even worse when an exacerbation has already occurred.

Cvietusa et al., in a study of 1697 adult patients for 12 months after an asthma exacerbation, showed that adherence to treatment as measured by portion of days covered (PDC) improved significantly by 20% (from 0.44 to 0.53; *p* < 0.001) after an asthma exacerbation and even more by 27% (from 0.45 to 0.57; *p* < 0.001) in patients who had more than one exacerbation since the initial exacerbation. In addition, among patients who were seen by a specialist after an exacerbation, there was a significant improvement in PDC (0.17 mean change). This emphasizes the role of the physician in managing adherence to asthma but also shows that patients often just face the negative impact of an exacerbation and start to become adherent to therapy [19].

### 4.6. What Should We Do?

As we have noted, there are countless methods for monitoring adherence to asthma treatment, and the answer to the question of which is the best way to quantify adherence is still far from being answered. However, several guidelines should be kept in mind, and the method chosen should be adapted to the patient as well as to the possibilities of the health units in which the specialist works.

First of all, increasing patient education and awareness of the importance of adherence to treatment is essential for asthma control, and at the same time, the physician must make it a priority to assess adherence at each consultation. Also, information campaigns and social support, even through telemedicine, can bring significant improvements.

In addition, the physician can quickly and inexpensively perform specific adherence questionnaires, which, in conjunction with usual clinical, functional, and biological status, can provide a first impression of adherence. With these usual methods, non-adherent or probably non-adherent patients can be identified, in which case objective methods such as specific biomarkers depending on the type of treatment followed or electronic monitoring devices should be used. However, at present, due to financial problems, objective methods of adherence monitoring in asthma are still an elusive ideal in many medical centers, but will certainly represent important perspectives in the future.

The advantages and disadvantages of the most representative adherence monitoring methods in asthma discussed in this review are summarized in Table 2.

## 5. Oral Corticosteroids and the Implications of Non-Adherence

Treatment with oral medication, especially OCS, is frequently used in severe asthma but is replaced nowadays gradually by other types of therapy because of the numerous side effects reported. Oral corticosteroids given for prolonged periods decrease patients’ immunity and have an increased risk of psychosis, hypertension, adrenocortical insufficiency, and osteopenia [45,137,138,139].

Oral corticosteroids used in high doses over a long period have been associated with a 63% increased risk of mortality and a two-fold increased risk of vertebral fracture. Analyzing records comprising the OCS prescriptions of 151,760 patients in a cohort study revealed an association between the dose administered and the body’s response associated with increased risk of morbidity, mortality, and hospitalization [140].

In a Belfast study, which objectively tested adherence to OCS with a cortisol/prednisolone test in 51 patients, 49% of patients were not adherent. However, only when confronted with objective measurements did patients recognize that they had inadequate adherence. Another study monitoring OCS adherence in 28 patients conducted in London shows that 32% of subjects had undetectable prednisolone or cortisol, which was not suppressed despite being prescribed ≥15 mg prednisolone daily. In addition, it was observed in these two studies that this problem is not routinely recognized, as most enrolled patients were referred by other medical specialists, favoring inappropriate escalation to prolonged OCS or costly biologic therapies [127].

In addition, a cohort study U-BIOPRED, which enrolled 166 patients, used the Medication Adherence Report Scale (MARS) questionnaire to test adherence to OCS in severe asthma, which was correlated with objective urine prednisolone measurement, found that in 53% of cases, no agreement between the methods was detected, emphasizing once again the limitations of self-reporting [141].

Although the current trend is to avoid the adverse effects of OCS by early initiation of costly biological therapies, this is often not unjustified [96,142]. A cross-sectional study of Dutch patients diagnosed with uncontrolled or severe asthma shows that even though the use of high-dose OCS was high (29.5% of patients), 78.1% of these patients were wrong labeled as candidates for biologic therapy, as 47.4% were not adherent to ICS and 30.7% had poor inhalation technique or were susceptible to poor adherence. Only 21.9% were considered definitive candidates for biologic therapy. Thus, this study demonstrates that in 4 out of 5 patients, adherence to ICS and/or technique was suboptimal, highlighting the importance of the clinician in optimizing inhaled therapy to reduce the need for OCS and minimize the prescription of inappropriate, costly biologic therapies [142].

## 6. Biologic Asthma Therapies and the Implications of Non-Adherence

Genetic and immunologic research advances have defined asthma’s molecular mechanisms today [125,128,143,144]. New treatments have been developed, including biologic agents (monoclonal antibodies), which are safe and effective therapies for reducing airway inflammation [96,128,145]. However, before initiating biological therapy, treatment adherence must be strictly controlled. The frequency and number of injection administrations vary substantially from agent to agent and are strongly associated with adherence to these treatments [146].

Omalizumab is a humanized monoclonal antibody that binds to the Fc fragment of IgE and inhibits IgE binding to the receptor on mast cells [147]. Patient adherence to Omalizumab is high in some studies, as a significant improvement in patients with allergic asthma has been observed in reducing symptoms and exacerbation episodes [147,148,149].

Research shows that the adherence concerning treatment duration of the oldest monoclonal antibody produced, Omalizumab, in 196 patients is 90.7%. Increased treatment adherence in this study was associated with a better ACT score, older age, and 14-day timing [150]. This study found good adherence in 88% of patients treated with Omalizumab for less than 2 years, and treatment was missed in less than 10% of cases. In addition, an increase in adherence of up to 100% was observed in patients treated with Omalizumab for more than 4 years [150,151]. Another study of 3058 patients shows an average PDC of 74% for Omalizumab and a PDC of ≥80% in 60.6% of patients [149,151].

A 12-month US study of 3058 patients diagnosed with asthma initiated on Omalizumab therapy over 12 months shows that 60.6% of patients were adherent, defined by a PDC ≥ 80%. In contrast, 36.9% discontinued therapy, of whom only 26.3% switched to a new asthma therapy. In addition, it was observed that adherence was better when the number of physician visits for evaluation was higher. Also, patients with associated rhinitis as a comorbidity and a high number of medical visits had significantly lower odds of discontinuing therapy and increased odds of adherence to Omalizumab than patients without rhinitis. Finally, higher rates of adherence to Omalizumab were observed in patients using ICS, whereas the use of OCS was not significantly associated with adherence to this therapy [149].

Another 12-month study of 3496 patients on adherence to Mepolizumab (an interleukin-5 antagonist monoclonal antibody) according to PDC showed an adherence of 61.5%, with an average PDC of 70% [151].

In some cases, increased quality of life causes asthmatics to give up biological therapy or not follow the therapeutic regimen prescribed by the specialist. The SHAMAL study of 208 patients on biologic therapy with benralizumab (anti-interleukin-5 receptor α) shows that patients with controlled asthma on biologic treatment can significantly reduce inhaled therapy [152]. However, this dose reduction should be made at the indication and under the supervision of the physician, not deliberately by the patient.

In addition to monitoring behavior to biologic therapy, adherence to ICS should also be monitored among these patients, as some patients with evident early clinical benefit because of biological therapies may deliberately reduce their inhaled corticosteroid medication, which may lead to increased FeNO and exacerbations. This biomarker may indicate poor adherence. Thus, checking the inhalation technique and encouraging the use of background medication are particularly important for these patients [153].

Concerning the administration, a qualitative survey of 75 patients conducted via online forums shows that many patients on biological therapy report that they never forget to administer their injection, and among the reminder strategies used are diaries, smartphones, and e-mail. Affirmatively, patients who have forgotten to take their biologic claim that the delay was from a few hours to a few days because they had other concerns [154].

Another US study of 5319 patients initiated on biologic therapy for asthma shows that adherence, as quantified by the mean PDC in the first 6 months of biologic medication (PDC = 0.76), was higher than the mean PDC of ICS, both before 6 months (PDC = 0.44) and after 6 months (PDC = 0.4) of starting asthma biologics. Additional statistics for each individual biologic show that in the first 6 months, the highest mean adherence by average PDC was with Dupilumab (98.1%), followed by Reslizumab (92%), Mepolizumab (88.5%), Benralizumab (80.7%), and Omalizumab (71.8%) [155].

The CHRONICLE study described adherence to biological therapy in asthma, assessing the PDC in the first 52 weeks after initiation in 2117 patients and analyzing 61,176 administrations. It finds that patients diagnosed with severe asthma are predominantly adherent to biologic therapy, showing a median PDC of 86.8%, with 73.84% of patients having a PDC ≥ 75%. The best adherence is observed in patients receiving Mepolizumab (82.1%), Reslizumab (78.4%), and Benralizumab (78.6%), while Omalizumab (73.7%) and Dupilumab (54.4%) showed poorer results [156].

In general, current estimates of adherence to biologic treatments in severe asthma (SA) state that about 60% of patients have good adherence to treatment based on PDC, results like previous assessments [149,151,156]. When referring to adherence to biologics, the frequency between administrations and the place and mode of administration of each biologic agent should be considered; as currently, there are studies that show that taking biologic therapies at longer intervals is associated with better adherence [151,157]. More and more patients are adopting self-administration at home, thus creating an environment challenging to supervise the specialist [156]. Therefore, self-administration should only be considered when beneficial [88].

Moreover, in the CHRONICLE study, the proportion of systemic corticosteroid (SCS) use prior to 12 months of therapy initiation was one of the variable enrollment characteristics, being 22% for Reslizumab, 17% for Dupilumab, 16% for Benralizumab, 16% for Mepolizumab, and 7% for Omalizumab. At the same time, this study shows that Dupilumab, compared to the other monoclonal antibodies, was predominantly (84%) administered by the patient or a member of the patient’s family at home; subsequently, during the study, this trend was increasing for the other biologics, reaching approximately 50% of biologic administrations by the end of 2021. Dupilumab had the lowest median PDC (83%), likely due to high home administration. In addition, the highest delay in administration was observed in Dupilumab, suggesting low adherence, whereas the lowest delay was identified in Benralizumab, suggesting higher adherence. These findings suggest that the more infrequently the biologic is administered, the more it is preferred by patients [156].

Data on biological treatment modification are still limited. Studies of severe asthma therapies, including 3531 patients from 11 countries (2015–2020), show that 79% of patients continued with their initial biologic treatment, 10.2% stopped biologic therapy, and 10.8% switched to a different therapeutic agent. Among the most common reasons for changing or even stopping biological treatment were lack of efficacy or adverse effects [158]. Another reason why asthmatic patients may become non-adherent to immunotherapies is the lack of symptoms after a while. The patient increases functional lung capacity, reintegrates into society, improves quality of life, and no longer complies with specialist recommendations.

**Figure 2:**
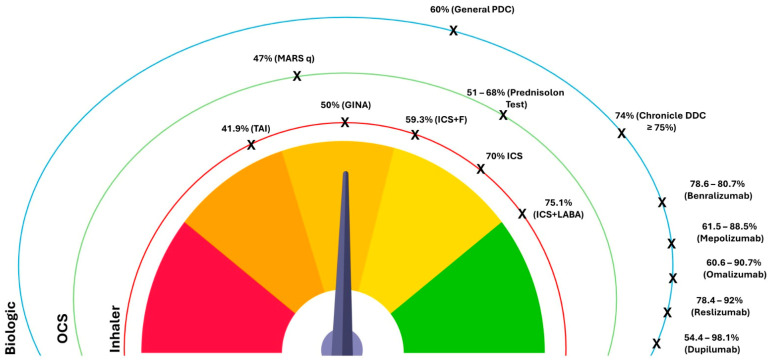
Quantification of adherence to different types of asthma treatment across reviewed studies.

The quantification of adherence to different types of asthma treatment according to the studies used in this review, regardless of the method of measurement, is graphically depicted in Figure 2.

## 7. Conclusions

This narrative review provides an overview of treatment adherence in asthma and highlights the critical role of inhaler devices, patient education, and the integration of electronic monitoring technologies in enhancing treatment adherence. Addressing common errors in inhaler use and leveraging real-time feedback from electronic devices can significantly improve patient outcomes. Moreover, when available, objective methods, such as biomarkers or EMDs, should be considered and tailored for each individual patient to create personalized treatment strategies that may offer promising avenues for long-term asthma adherence management.

Achieving optimal asthma control necessitates ongoing collaboration between healthcare providers and patients, with targeted engagement in asthma adherence, focused on personalized care, continuing education, and innovative technologies to do everything possible to prevent exacerbations.

## Figures and Tables

**Table 1:** Overview data on adherence in asthma.

Overview
Non-adherence to treatment was the most important modifiable factor in preventing asthma deaths
After an asthmatic exacerbation, patients become 20% more adherent to asthma treatment
Up to 70–80% of asthma patients do not use their inhalers correctly
Estimates of adherence in asthma are between 22% and 78%
Objective measurements showed that self-assessment overestimated adherence in asthma by more than 50%
Self-reported non-adherence is an essential qualitative indicator of poor adherence
Inhalation technique errors, exacerbations, and costs of care are directly correlated with age, previous training, comorbidities, and educational and socioeconomic status.

**Table 2:** Advantages and disadvantages of the most representative adherence monitoring methods in asthma.

Monitoring Method	Advantages	Disadvantages
Adherence monitoring questionnaires	High clinical applicability Low costs Can provide information quickly	Subjective measurement May provide incomplete/false answers Prone to recall bias
Prescription monitoring	Objectives, based on health system data Can show long-term trends	Does not reflect the actual use of the medicine
Biomarker monitoring	Provides objective and quantifiable data	Costly Limited to specific cases
Electronic reminder systems	Can provide maintenance of treatment routine	It may be ignored by patients Requires technology education
Electronic monitoring devices	Real-time feedback, easy to use Improves patient self-management	Costly Requires technology education
Radiological methods	Objectively confirm the amount of inhaled particles in humans	Costly radiation exposure Limited long-term use
